# Obituary

**Published:** 1909-05-15

**Authors:** 


### OBITUARY.

### ALLISON WRIGHT HARLAN, D.D.S., M.D., A.M.

Dr. Harlan was born November 15th, 1850, in Juliett, Harlan Co., Indiana, and died March 6th, 1909, in New York City.

Dr. Harlan began the study of dentistry in 1869 with Drs. Kilgore and Helms, in Indianapolis, and moved to Chicago and began practice for himself in 1870. In the same year he married Miss Bessie Muirson, of Indiana. In 1878 and 79 he attended the Ohio College of Dental Surgery, graduating in 1879.

Dr. Harlan has always taken a large interest in the work of the profession, and it would be difficult to summarize the tremendous efforts made by him to advance his profession. He was an indefatigable worker and enjoyed an extensive practice for many years in Chicago. He established the Dental Review and was for many years its editor. He did much to make Chicago the recognized center of dental activity by his energetic work in professional matters. For many years he was Professor of Dental Materia Medica and Therapeutics in the Chicago Dental College and became an authority on this subject. He was always active in the State and National Dental Societies and was honored with the highest offices in them. He also did much to interest the Foreign dentists in the great Inter-National work which has done so much to bring about cordial international relations. He was a man with a great memory and had in his mind all the important historical events of his profession. He was well known all over the world as a man deeply in love with his profession. He had a large acquaintance and a host of friends who will mourn his untimely death. He moved to New York City in 1903 to engage in the practice of Oral Hygiene as a specialty in which he was very skillful and successful. He died from an operation for a ventral hernia, March 6th, and was burried in Kensico cemetery, New York.

